# UCHL1-HIF-1 axis-mediated antioxidant property of cancer cells as a therapeutic target for radiosensitization

**DOI:** 10.1038/s41598-017-06605-1

**Published:** 2017-07-31

**Authors:** Ryota Nakashima, Yoko Goto, Sho Koyasu, Minoru Kobayashi, Akiyo Morinibu, Michio Yoshimura, Masahiro Hiraoka, Ester M. Hammond, Hiroshi Harada

**Affiliations:** 1 Department of Radiation Oncology and Image-applied Therapy, Kyoto University Graduate School of Medicine, 54 Shogoin Kawahara-cho, Sakyo-ku, Kyoto 606-8507 Japan; 2 Laboratory of Cancer Cell Biology, Department of Genome Dynamics, Radiation Biology Center, Kyoto University, Yoshida Konoe-cho, Sakyo-ku, Kyoto 606-8501 Japan; 3 CRUK/MRC Oxford Institute for Radiation Oncology, Department of Oncology, University of Oxford, Oxford, OX3 7DQ United Kingdom; 4 Precursory Research for Embryonic Science and Technology (PRESTO), Japan Science and Technology (JST), 4-1-8 Honcho, Kawaguchi, Saitama 332-0012 Japan

## Abstract

Hypoxia-inducible factor 1 (HIF-1) has been recognized as an important mediator of the reprogramming of carbohydrate metabolic pathways from oxidative phosphorylation to accelerated glycolysis. Although this reprogramming has been associated with the antioxidant and radioresistant properties of cancer cells, gene networks triggering the HIF-1-mediated reprogramming and molecular mechanisms linking the reprogramming with radioresistance remain to be determined. Here, we show that Ubiquitin C-terminal hydrolase-L1 (UCHL1), which we previously identified as a novel HIF-1 activator, increased the radioresistance of cancer cells by producing an antioxidant, reduced glutathione (GSH), through HIF-1-mediated metabolic reprogramming. A luciferase assay to monitor HIF-1 activity demonstrated that the overexpression of UCHL1, but not its deubiquitination activity-deficient mutant (UCHL1 C90S), upregulated HIF-1 activity by stabilizing the regulatory subunit of HIF-1 (HIF-1α) in a murine breast cancer cell line, EMT6. UCHL1 overexpression induced the reprogramming of carbohydrate metabolism and increased NADPH levels in a pentose phosphate pathway (PPP)-dependent manner. The UCHL1-mediated reprogramming elevated intracellular GSH levels, and consequently induced a radioresistant phenotype in a HIF-1-dependent manner. The pharmacological inhibition of PPP canceled the UCHL1-mediated radioresistance. These results collectively suggest that cancer cells acquire antioxidant and radioresistant phenotypes through UCHL1-HIF-1-mediated metabolic reprogramming including the activation of PPP and provide a rational basis for targeting this gene network for radiosensitization.

## Introduction

Significant technological improvements in the field of radiation therapy, such as three-dimensional conformal radiation therapy (3D-CRT), intensity-modulated radiation therapy (IMRT)^1^, and image-guided radiation therapy (IGRT), have facilitated both dose escalations to target volumes and dose-sparing to normal tissues^2^. As a result radiation therapy has become increasingly important in cancer therapy and is now applied globally for a growing number of cancer patients^2, 3^. However, patients often suffer from local tumor recurrence after radiation therapy due to the presence of radioresistant cancer cells in malignant solid tumors^4–6^. Accumulating evidence has demonstrated that several factors, such as the cell cycle status, DNA damage repair activity, oxygen-availability, and pH, intricately influence one another and eventually lead to the radioresistant properties of cancer cells^6–12^. It has been widely accepted that the so-called chemo-radiotherapy, a combination of radiation therapy with chemotherapeutic agents, which appropriately controls these complexities, is a rational strategy to overcome radioresistance^5, 10^. Among the intrinsic and extrinsic factors behind the radioresistance of cancer cells, gene networks responsible for the production of antioxidants have drawn considerable attention in recent years^6, 13^.

The growth advantage of cancer cells is known to be attributed to the unique glucose metabolic pathway, the so-called Warburg Effect, which is characterized by the production of ATP through accelerated glycolysis rather than mitochondrial oxidative phosphorylation, not only under hypoxic but also normoxic conditions^6, 14, 15^. Glucose-6-phosphate, an intermediate metabolite of glycolysis, is the initial substrate of the pentose phosphate pathway (also known as the phosphogluconate pathway and hexose monophosphate shunt), which generates NADPH and pentoses (5-carbon sugars) as well as ribose-5-phosphate^16–18^. A recent study demonstrated that the pentose phosphate pathway is associated with the radioresistance of cells^19^ because its byproduct, NADPH, is essential for the production of an antioxidant, reduced glutathione (GSH), from glutathione-S-S-glutathione (GSSG), and because ribose-5-phosphate is used in the de-novo synthesis of nucleotides, which are essential for repairing DNA damage. However, a gene network triggering the reprogramming of carbohydrate metabolism and the subsequent pentose phosphate pathway has yet to be fully elucidated.

Hypoxia-inducible factor 1 (HIF-1), which is known as a master regulator of the cellular adaptive response to hypoxia^20, 21^, has been recognized as an important player in the metabolic reprogramming of cancer cells^22–24^. HIF-1 functions as a heterodimeric transcription factor composed of an α (HIF-1α) and β (HIF-1β) subunit, and its activity is known to be mainly dependent on the expression levels and transactivation activity of HIF-1α^20, 25^. HIF-1α expression has been reported to be regulated at multiple levels: at transcriptional initiation stimulated by phosphatidylinositol 3 kinase-Akt/protein kinase C/histone deacetylase (PI3K-Akt/PKC/HDAC) signaling^26^, at translational initiation controlled by PI3K/Akt/mammalian target of rapamycin (mTOR) signaling^27^, and at proteolysis mediated by prolyl hydroxylation at P402 and P564 of HIF-1α by prolyl-4-hydroxylases (PHDs)^20, 28–30^ and subsequent ubiquitination by von Hippel Lindau (VHL)-containing E3 ligase^31, 32^. On the other hand, the transactivation activity of HIF-1α is regulated through asparaginyl hydroxylation at N803 by factor inhibiting HIF-1 (FIH-1)^20, 33^. Among these regulatory steps, the degradation of HIF-1α protein is mainly responsible for the normoxia-dependent inactivation/hypoxia-dependent activation of HIF-1.

Because of the highly divergent functions of HIF-1 in the malignant progression of cancers, gene networks, which potentially upregulate HIF-1, have drawn considerable attention in cancer research^6, 34–38^. Establishing a sophisticated genetic screening system, we recently identified novel upstream activators of HIF-1, including ubiquitin C-terminal hydrolase L1 (UCHL1)^39^, isocitrate dehydrogenase 3 (IDH3)^40^, and lymphocyte antigen 6 complex, locus E (LY6E)^41^, and revealed their functions in the malignant progression of tumors. HIF-1 is associated with not only carbohydrate metabolic reprogramming^23, 24^ but also radioresistance of cancer cells^6, 10, 11, 42^. However, both the gene network triggering HIF-1-mediated reprogramming and the molecular mechanism linking the reprogramming with radioresistance remain to be determined.

In the present study, we focused on the UCHL1-HIF-1 axis and investigated whether it induced the metabolic reprogramming, antioxidant property, and radioresistant phenotype of cancer cells using murine breast cancer-derived EMT6 cells.

## Results

### UCHL1 deubiquitinates HIF-1α protein and upregulates HIF-1 activity in murine breast cancer-derived EMT6 cells

**Figure 1:**
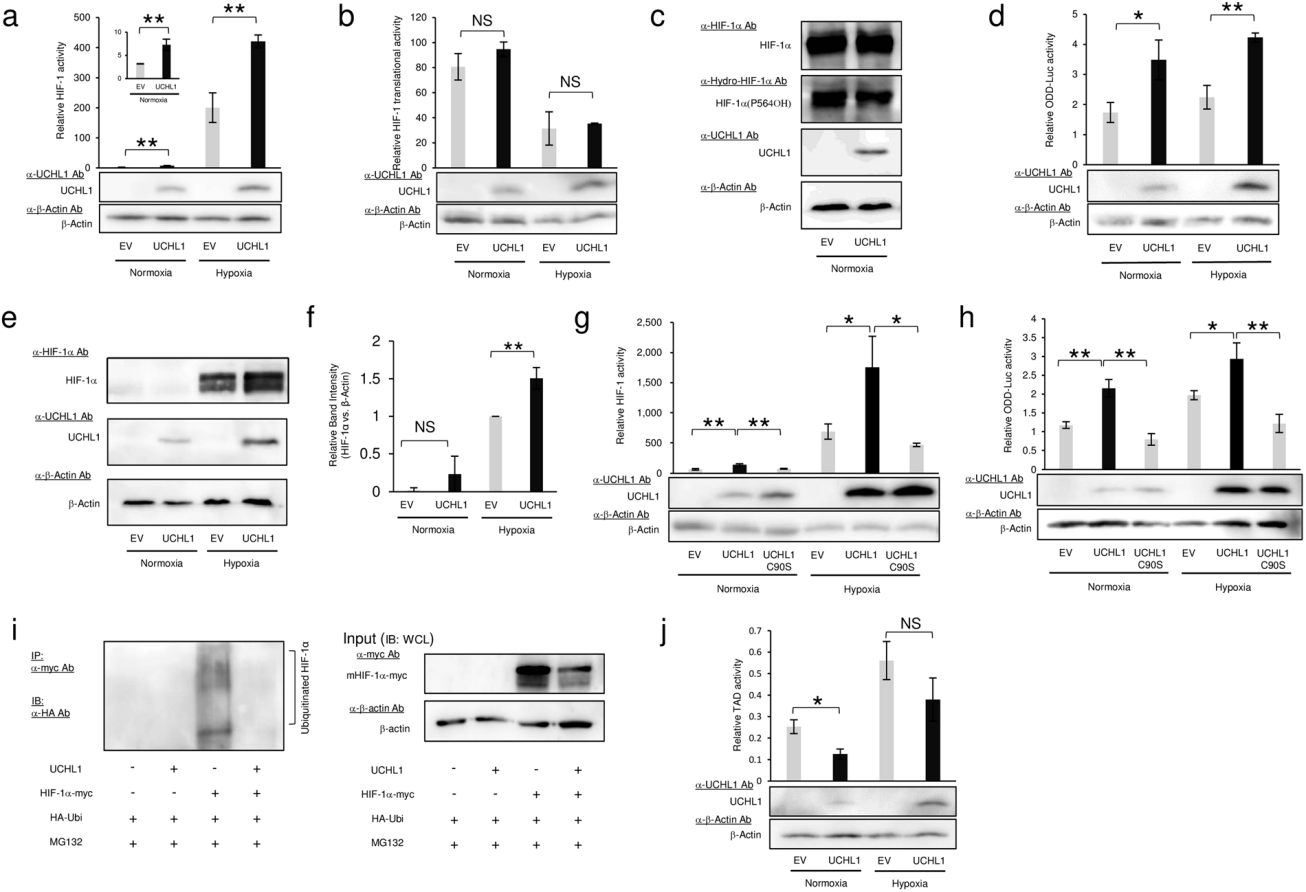
Importance of the deubiquitinating activity of UCHL1 in the upregulation of HIF-1 activity in murine breast cancer EMT6 cells. (**a–h**) EMT6 cells were transfected with either pcDNA4/UCHL1 (UCHL1) or its empty vector (EV), cultured under the indicated oxygen conditions, and subjected to both the luciferase assay using a co-transfected reporter plasmid, p5HRE-luc (**a**,**g**), pGL3/HIF-1α-5′UTR-Luc (**b**), or pGL3/ODD-luc (**d**,**h**), and Western blotting using the indicated antibodies (**a–h**). The relative intensity of the bands in Fig. 1e (HIF-1α vs. β-actin) was quantified using ImageJ (**f**). (**i**) Cells transiently transfected with both pMT132 and either pcDNA4/myc-His A (EV) or pcDNA4A/HIF-1α-myc were additionally transfected with pcDNA4/myc-His A (EV) or pcDNA4/UCHL1 (UCHL1), and cultured with MG132 (10 μM) for 6 h. Ubiquitinated HIF-1α was immunoprecipitated using an anti-myc antibody and detected with an anti-HA antibody (left). One-twentieth of the whole cell lysate was subjected to Western blotting with the indicated antibodies as input controls (right). (**j**) EMT6 cells were cotransfected with four kinds of plasmids: pG5H1bLuc containing five Gal4-binding sites upstream of an adenovirus E1b promoter and firefly luciferase CDS^33, 50^, pRL-SV40 as an internal control for normalization, pcDNA6/Gal4 DBD-HIF-1αTAD P564A, and either pcDNA4/UCHL1 (UCHL1) or its EV. Cells were cultured under normoxic or hypoxic conditions and subjected to the luciferase assay to evaluate the transactivation activity of HIF-1α TADs. All results (except for **c** and **e**) are presented as means ± *s*.*d*. n = 3. *P < 0.05. **P < 0.01. NS, not significant (Student’s t-test).

Because HIF-1 activity is known to be regulated at multiple steps, we first aimed to identify the key regulatory mechanism in the UCHL1-mediated activation of HIF-1 in EMT6 cells. First, we performed a luciferase assay using the *5HRE*-*luc* reporter gene^43^, which expresses luciferase bioluminescence in a HIF-1-dependent manner, in order to test whether UCHL1 enhanced HIF-1 activity in EMT6 cells. We transfected the cells with the reporter gene and UCHL1 expression vector, cultured them under normoxic or hypoxic conditions, and performed the luciferase assay. The forced expression of UCHL1 significantly enhanced HIF-1 activity regardless of the oxygen conditions (Fig. 1a). We next examined the impact of UCHL1 overexpression on the translational initiation of HIF-1α protein. We employed the *HIF*-*1α 5*′*UTR*-*luc* reporter gene^39^, in which a pyrimidine tract of HIF-1α 5′UTR was inserted between the constitutively active CMV promoter and luciferase coding sequence^27, 39^, and confirmed that UCHL1 exhibited no influence on the translational initiation of the HIF-1α gene (Fig. 1b).

Because HIF-1 activity is markedly influenced by the balance between the degradation and stabilization of HIF-1α protein, we next analyzed the possibility that UCHL1 affected this balance. Western blotting using an antibody to detect the hydroxylated proline residue 564 of HIF-1α, demonstrated that the hydroxylation, which could be detected in the presence of the proteasome inhibitor MG132 in an oxygen- and PHD-dependent manner, was not affected by the forced expression of UCHL1 (Fig. 1c). On the other hand, by utilizing the *SV40p*-*ODD*-*Luc* reporter gene^44^, which expressed a fusion protein composed of a HIF-1α ODD domain (HIF-1α 548–604) and luciferase from the constitutively active SV40 promoter, we confirmed that UCHL1 was able to actively stabilize the ODD-luciferase fusion protein (Fig. 1d). This finding was supported by Western blotting for HIF-1α protein; namely, expression levels of HIF-1α protein were significantly increased under hypoxic conditions when the UCHL1 expression vector was introduced into the cells (Fig. 1e and f). However, whether this also occurred under normoxic conditions as well remained unclear, as the basal HIF-1α expression levels were below the detection limits of Western blotting (Fig. 1e and f). Since UCHL1 has been recognized as a deubiquitinating enzyme^45^, we tested whether its ubiquitinating activity is critical for the stabilization of HIF-1α protein. The luciferase assay using the *5HRE*-*luc* reporter gene showed that a catalytically inactive mutant of UCHL1, UCHL1 C90S^46^, failed to upregulate HIF-1 activity (Fig. 1g). The luciferase assay using the *SV40p*-*ODD*-*Luc* reporter gene also demonstrated that UCHL1 C90S overexpression did not stabilize the ODD-fusion protein (Fig. 1h). In addition, when we tested whether UCHL1 influenced the ubiquitination status of HIF-1α by performing an immunoprecipitation experiment, the forced expression of UCHL1 markedly decreased the amount of ubiquitinated HIF-1α (Fig. 1i). Finally, although we examined the function of UCHL1 in the upregulation of the transactivation activity of HIF-1 by utilizing another luciferase assay system^40^, the forced expression of UCHL1 had no effect on it (Fig. 1j). These results collectively indicate that UCHL1 stabilizes HIF-1α protein, and elicits HIF-1 activity in a deubiquitinating activity-dependent manner in EMT6 cells.

### The UCHL1-HIF-1 axis induces reprograming of the glucose metabolic pathway and subsequent production of an antioxidant, GSH

**Figure 2:**
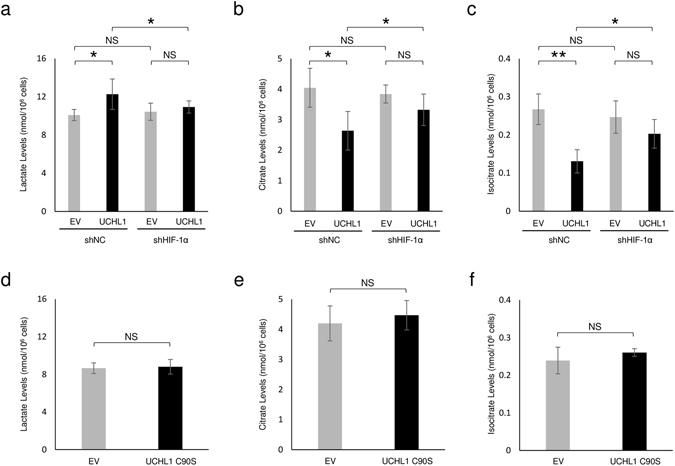
Metabolite levels in UCHL1-overexpressing murine breast cancer EMT6 cells. (**a**–**f**) Metabolites extracted from EMT6 cells transfected with the indicated expression vector for none (EV: **a**–**f**), UCHL1 (**a**–**c**), or UCHL1 C90S mutant (**d**–**f**), and with the indicated shRNA for HIF-1α (shHIF-1α: **a**–**c**) or scramble negative control (shNC: **a**–**c**) were subjected to quantitative analyses of [^13^C_3_]lactate (**a**,**d**), [^13^C_2_]citrate (**b**,**e**), and [^13^C_2_]isocitrate levels (**c**,**f**). Means ± *s*.*d*. n = 3. *P < 0.05, **P < 0.01. NS, not significant (Student’s t-test).

Although HIF-1 has been reported to induce reprogramming of the glucose metabolic pathway from mitochondrial oxidative phosphorylation to glycolysis^23, 24^, it remains unclear whether UCHL1 acts as a molecular trigger for this switch. To examine this possibility, we analyzed the influence of UCHL1 overexpression on the choice of metabolic pathway by quantifying levels of an end-metabolite of glycolysis, lactate, and primary metabolites in the TCA cycle, citrate and isocitrate (Fig. 2). LC/MS-based metabolite analyses demonstrated that the overexpression of UCHL1 significantly increases the flux of metabolism from glucose to lactate (Fig. 2a) and, on the other hand, it decreased glucose metabolism to both citrate and isocitrate (Fig. 2b and c). The observed changes in flux were partially but significantly suppressed by silencing the expression of HIF-1α. Importantly, the suppressive impacts of HIF-1α silencing were detected in the presence of UCHL1 expression, but not in its absence (Fig. 2a–c). All of the data suggest an important role of UCHL1 as a trigger for the HIF-1-dependent metabolic reprogramming from mitochondrial oxidative phosphorylation to aerobic glycolysis (Fig. 2a–c).

**Figure 3:**
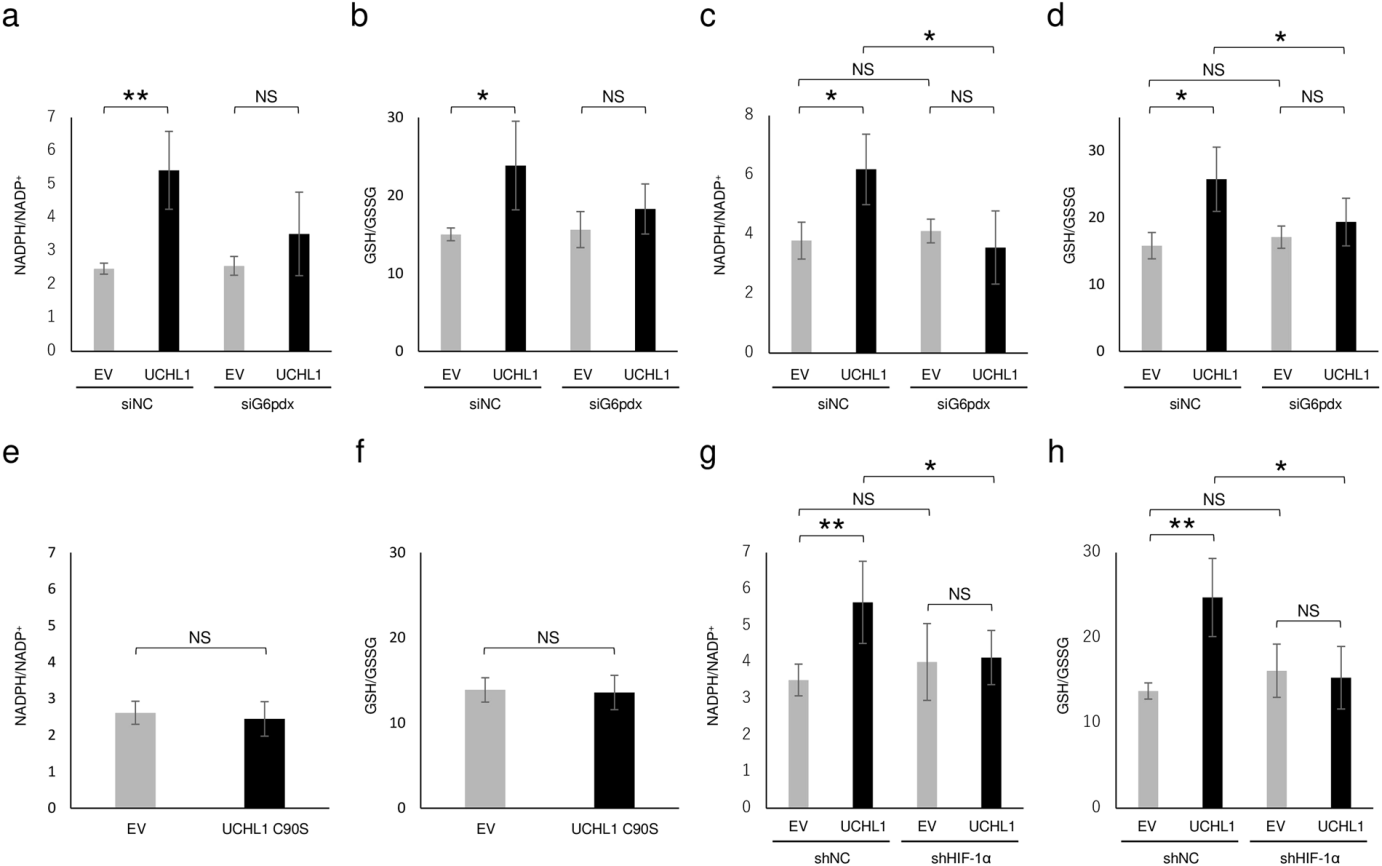
Antioxidant levels in UCHL1-overexpressing murine breast cancer EMT6 cells. (**a**–**h**) EMT6 cells transfected with the indicated expression vector for none (EV; **a**–**h**), UCHL1 (**a**–**d**,**g**,**h**), or UCHL1 C90S (**e**,**f**), or for shRNA against HIF-1α (shHIF-1α; **g**,**h**) or scramble shRNA (shNC; **g**,**h**), or with the indicated siRNA for G6pdx (siG6pdx; **a**–**d**) or scramble siRNA (siNC; **a**–**d**) were subjected to quantitative analyses of the ratio of NADPH to NADH^+^ (**a**,**c**,**e**,**g**) and GSH to GSSG (**b**,**d**,**f**,**h**) using the NADP/NADPH Quantification Colorimetric Kit (**a**,**e**,**g**), the NADP/NADPH-Glo Assay Kit (**c**), the Glutathione Colorimetric Assay Kit (**b**,**f**,**h**), and the GSH/GSSG-Glo Assay Kit (**d**). Means ± *s*.*d*. n = 3. *P < 0.05, **P < 0.01. NS, not significant (Student’s t-test).

We next tested whether reprogramming was accompanied by activation of the pentose phosphate pathway and whether this led to the antioxidant properties of cancer cells. Quantitative analyses of the ratio of NADPH to NADP^+^ (NADPH/NADP^+^) and that of GSH to GSSG (GSH/GSSG) revealed that the aberrant overexpression of UCHL1 increased intracellular levels of both NADPH and GSH (Fig. 3a and b). The increases in both NADPH and GSH were partially but significantly suppressed by silencing the expression of a key element of the pentose phosphate pathway, the glucose-6-phosphate dehydrogenase X-linked (G6pdx) gene (Fig. 3a and b). In agreement with these results, luciferase assay-based quantification experiments also confirmed that overexpression of UCHL1 significantly increased the intracellular levels of both NADPH and GSH in a pentose phosphate pathway-dependent manner (Fig. 3c and d). On the other hand, forced expression of the UCHL1 C90S mutant neither induced the carbohydrate metabolic reprogramming nor increased the levels of antioxidant GSH (Figs 2d–f and 3e and f). Moreover, the UCHL-dependent increases in the levels of both NADPH and GSH were almost completely suppressed by silencing the HIF-1α gene (Fig. 3g and h). Taken together, these results strongly suggest the possibility that activation of the UCHL1-HIF-1 axis causes the production of the antioxidant GSH by reprograming the glucose metabolic pathway and stimulating the pentose phosphate pathway.

### The UCHL1-HIF-1 axis functions in the induction of the radioresistant phenotype of cancer cells

**Figure 4:**
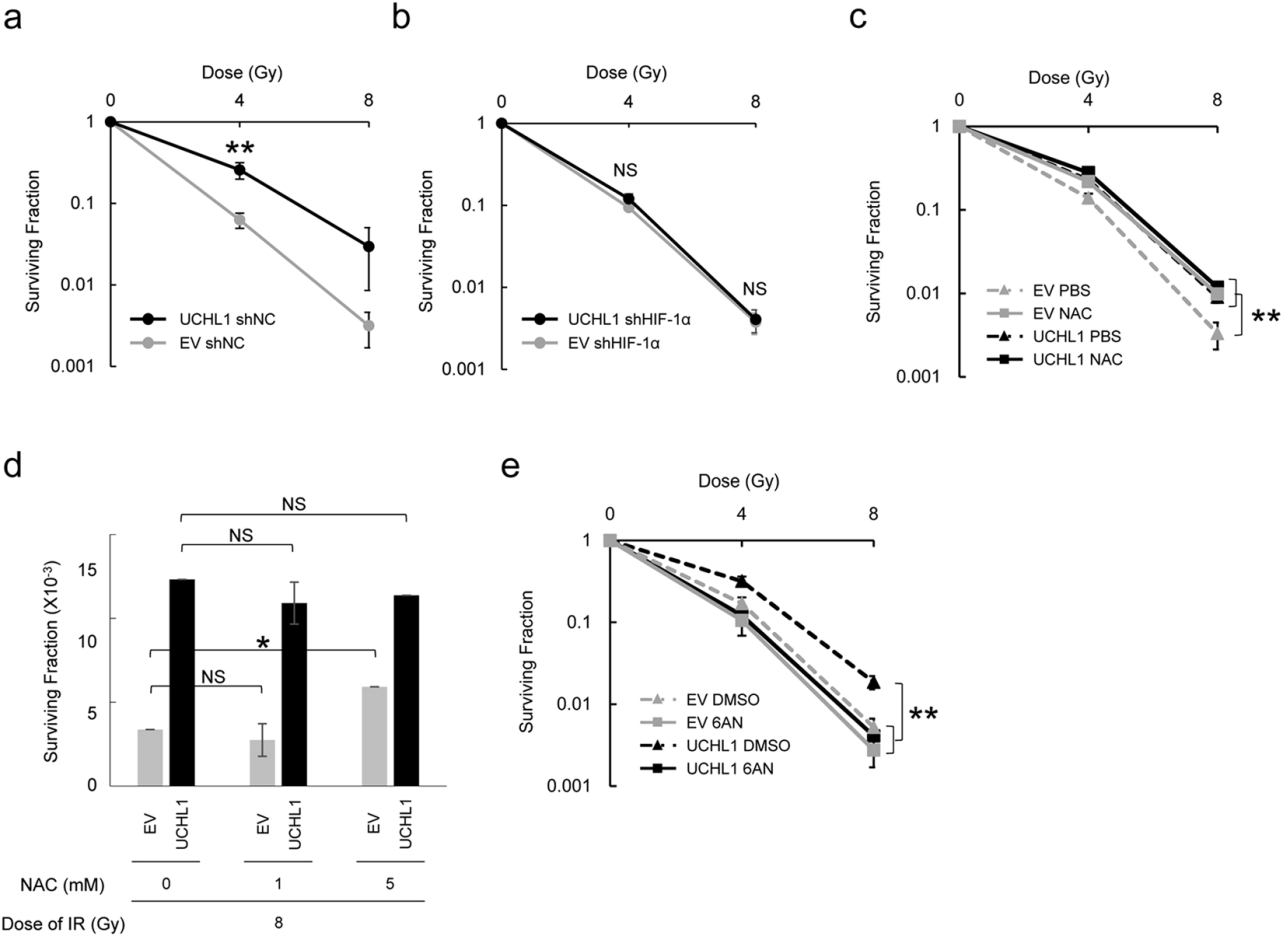
Influence of the UCHL1-HIF-1 axis on radiosensitivity of EMT6 cells. (**a**) The clonogenic survival assay with the indicated dose of X-irradiation was performed using EMT6 cells transfected with either the UCHL1 expression vector (EMT6/EF-Luc/shNC/UCHL1 cells: UCHL1) or its empty vector (EMT6/EF-Luc/shNC/EV cells: EV). (**b**) The clonogenic survival assay was performed using EMT6 cells transfected with the expression vector of a short hairpin RNA for the HIF-1α gene and with either the UCHL1 expression vector (EMT6/EF-Luc/shHIF-1α/UCHL1 cells) or its empty vector (EMT6/EF-Luc/shHIF-1α/EV cells). (**c**,**d**) The clonogenic survival assay with the indicated doses of X-irradiation was performed using EMT6 cells transfected with either the UCHL1 expression vector (UCHL1) or its empty vector (EV) in the presence or absence of 5 mM (**c**) or the indicated concentrations (**d**) of NAC. (**e**) Clonogenic survival assay using EMT6 cells transfected with either the UCHL1 expression vector (EMT6/EF-Luc/shNC/UCHL1 cells: UCHL1) or its empty vector (EMT6/EF-Luc/shNC/EV cells: EV) in the presence or absence of a G6pdx inhibitor, 6AN (100 μM). Means ± *s*.*d*. n = 3. *P < 0.05, **P < 0.01 (Student’s t-test).

We then performed conventional *in vitro* clonogenic cell survival assays to investigate whether UCHL1 causes the radioresistance of cancer cells in a HIF-1-dependent manner. EMT6 cells were transfected with the UCHL1 expression vector or its empty vector as a negative control and subjected to various doses of X-irradiation. The surviving fraction, calculated as described previously^47^, demonstrated that the UCHL1 overexpression significantly increased clonogenic survival after X-irradiation (Fig. 4a). The dose of radiation needed to decrease the number of colonies by 90% (D_10_ value) was significantly increased after UCHL1 overexpression from 3.25 ± 0.08 to 5.54 ± 0.78 Gy in EMT6 cells (*P* < 0.05; Table 1). The UCHL1-mediated radioresistance was confirmed using another cell line, HeLa (Supplementary Figure 1). We conducted similar clonogenic survival assays after silencing HIF-1α expression in parallel with UCHL1 overexpression in order to determine whether the UCHL1-mediated radioresistance was dependent on HIF-1. We found that HIF-1 knockdown completely abrogated the radioresistance of EMT6 cells mediated by UCHL1, as expected (Fig. 4b and Table 1). D_10_ values were equivalent regardless of the expression levels of UCHL1 when HIF-1α expression was silenced; D_10_ values were 3.49 ± 0.12 and 3.64 ± 0.17 Gy in the EV and UCHL1 groups, respectively (*P* = 0.13; Table 1). In order to investigate whether the UCHL1-mediated radioresistance was attributable to the increased antioxidant property of cells, we performed an additional clonogenic cell survival assay with X-irradiation in the presence or absence of one of the most representative antioxidants, N-acetyl cysteine (NAC). We confirmed that, although the pH of the culture medium, which was originally 7.62 ± 0.03, decreased to 7.39 ± 0.03 at 6 min after NAC addition, it immediately returned to its original value, 7.60 ± 0.01, and remainded at that level until cells were treated with radiation; therefore, the change in pH did not directly influence the radiosensitivity of cells (Supplementary Figure 2). The surviving fraction was significantly increased by 5 mM NAC treatment in cases without UCHL1 overexpression (Fig. 4c and d). However, in cases with UCHL1 overexpression, the NAC treatment exhibited no further influence on cell survival (Fig. 4c and d), collectively suggesting that UCHL1 has the potential to induce the maximum cellular antioxidant effect on radiosensitivity. The UCHL1-dependent increase in cellular radioresistance was markedly decreased when intracellular levels of the antioxidant GSH were decreased by a G6pdx inhibitor, 6AN (Fig. 4e). All of these results strongly suggest that the aberrant overexpression of UCHL1 induces the antioxidant and radioresistant properties of cancer cells in a HIF-1- and G6pdx-mediated PPP-dependent manner.

**Table 1:** D_10_ values (The dose of radiation required to reduce the number of surviving colonies by 90%) in clonogenic survival assays of Fig. 4a and b.

Cell line	Expression Vector for	Silencing Vector for	D_10_ Value (Gy)
EMT6/EF-Luc/shNC/EV	None (empty vector)	Scramble shRNA	3.25 ± 0.08
EMT6/EF-Luc/shNC/UCHL1	UCHL1	Scramble shRNA	5.54 ± 0.78*
EMT6/EF-Luc/shHIF-1α/EV	None (empty vector)	HIF-1α shRNA	3.49 ± 0.12
EMT6/EF-Luc/shHIF-1α/UCHL1	UCHL1	HIF-1α shRNA	3.64 ± 0.17^NS^

*P < 0.05 vs. EMT6/EF-Luc/shNC/EV group.

NS: not significant vs. EMT6/EF-Luc/shHIF-1α/EV group.

## Discussion

In the present study, we found that the UCHL1-mediated activation of HIF-1 through the deubiquitination of HIF-1α protein induced the antioxidant and radioresistant properties of cancer cells by producing an antioxidant, GSH, through the so-called carbohydrate metabolic reprogramming and subsequent activation of the pentose phosphate pathway.

The luciferase assay using a deubiquitinating activity-deficient mutant of UCHL1 (C90S mutant) demonstrated that the ubiquitination activity of UCHL1 was essential to stabilize the ODD-fusion protein and upregulated HIF-1 activity in breast cancer-derived EMT6 cells. This result is consistent with a previous report that UCHL1 stabilized HIF-1α protein when VHL functioned as a key component of E3 ubiquitin ligase in the ubiquitination of HIF-1α protein^39^. In addition to such a molecular mechanism, we recently revealed the possibility that UCHL1 increases the expression levels of HIF-1α by upregulating the efficiency of the transcriptional initiation of the HIF-1α gene (data not shown). In order to fully elucidate the molecular mechanisms underlying the UCHL1-mediated upregulation of HIF-1 activity, further investigation is needed.

In the present study, although the constitutively active cytomegalovirus (CMV) promoter was exploited in the UCHL1 expression vector, UCHL1 protein levels were significantly increased under hypoxic conditions. These observations suggest that UCHL1 expression was upregulated at a post-transcriptional level, such as at mRNA stability levels, translational initiation levels, and/or protein stability levels. The increase in the UCHL1 protein levels under hypoxia might contribute to the rapid accumulation of HIF-1α protein in response to acute hypoxic stimuli. Alternatively, it may suggest the existence of a positive feedforward loop that boosts the accumulation of HIF-1α protein in the case that the hypoxia-dependent increase in the UCHL1 levels is HIF-1-dependent.

The radioresistance of cancer cells is influenced by various intrinsic and extrinsic factors, such as DNA damage repair activity, the cell cycle status, oxygen availability, and pH. Especially, gene networks, which induce the antioxidant property of cancer cells, have drawn considerable attention in recent years. Production of the most representative antioxidant, reduced glutathione (GSH), is mediated by multiple regulatory steps: cysteine uptake by the cystine/glutamate antiporter (system xc-)^48^, glutathione synthesis by the glutathione synthetase (GSS)^49^, and the reduction of glutathione-S-S-glutathione (GSSG) to GSH by glutathione-disulfide reductase (GSR), which uses NADPH as an electron donor^49^. Because NADPH is known to be provided as a byproduct of the pentose-phosphate pathway (PPP)^17, 18^, our result that UCHL1 overexpression increased radioresistance by producing GSH through the accelerated glycolysis and PPP is reasonable.

The UCHL1-dependent increase in the intracellular GSH levels was significantly suppressed by silencing the expression of a key molecule of the pentose phosphate pathway, the glucose-6-phosphate dehydrogenase X-linked (G6pdx) gene^17, 18^. Moreover, silencing the HIF-1α gene completely abrogated the UCHL1-mediated radioresistance of cancer cells. Based on these findings, our study provides an insight into a novel strategy targeting G6pdx and HIF-1α to overcome the UCHL1-dependent radioresistance of cancer cells.

Our clonogenic cell survival assays and the quantitative analysis of metabolite levels collectively demonstrated that the UCHL1-mediated radioresistance was at least in part dependent on the antioxidant property of cancer cells elicited by HIF-1. However, whether this mechanism is fully responsible for the radioresistance is questionable because HIF-1 is known to have numerous functions that potentially influence the radiosensitivity/radioresistance of cells, such as cell cycle regulation^6, 10, 11^. Further investigation is needed to fully understand the downstream effectors of the UCHL1-HIF-1 axis, which play critical roles in increasing the radioresistance of cancer cells.

## Methods

### Cell culture and reagents

EMT6, HeLa, and HEK293T were purchased from the American Type Culture Collection (Manassas, VA, USA). Cells were maintained at 37 °C in Dulbecco’s modified Eagle’s medium (DMEM). Media were supplemented with 10% FBS, 100 U/mL penicillin, and 100 µg/mL streptomycin. Cells were incubated in a well-humidified incubator with 5% CO_2_ and 95% air for the normoxic conditions or in a RUSKINN INVIVO_2_ 500 (Ruskinn) for the hypoxic conditions at <0.1% O_2_. Four lines of stable transfectants: EMT6/EF-Luc/shNC/EV, EMT6/EF-Luc/shHIF-1α/EV, EMT6/EF-Luc/shNC/UCHL1, and EMT6/EF-Luc/shHIF-1α/UCHL1 cells, were established previously^39^. Small inhibitory RNA (siRNA) against the *Mus musculus* glucose-6-phosphate dehydrogenase X-linked (G6pdx) gene was purchased from Invitrogen (Cat#4390771, Silencer Select: s66339-s66341).

### Plasmid DNA

To construct pcDNA4/UCHL1, the coding sequence of the human *uchl1* gene was amplified by PCR from the cDNA of HeLa cells and inserted between the EcoRV-XhoI sites of pcDNA4/myc-His A (Invitrogen), as described previously^39^. The plasmids pcDNA4/UCHL1 C90S^39^, p5HRE-Luc^43^, pGL3/ODD-Luc^44^, pGL3/HIF-1α-5′UTR-Luc^39^, and pcDNA6/Gal4-DBD-HIF-1 P564A^39^ were constructed as described previously. The hemagglutinin (HA)-tagged ubiquitin expression plasmid, pMT123, was described previously^32^. The plasmid pE1b-Luc was a kind gift from Prof. K Hirota (Kansai Medical University, Japan)^33, 40^.

### Luciferase assay and Western blotting

Twenty-four hours after cells (1 × 10^4^ cells/well in a 24-well plate for the luciferase assay and 1 × 10^5^ cells/well in a 6-well plate for Western blotting) were transiently transfected with the indicated plasmids using the Polyfection transfection reagent (QIAGEN), they were incubated under normoxic (20% O_2_) or hypoxic (<0.1% O_2_) conditions for the periods indicated in each figure legend, and lysed in 100 µL Passive Lysis Buffer (Promega) for the luciferase assay or 100 µL Cell Lytic Buffer (Sigma-Aldrich) for Western blotting. The luciferase assay was performed using the Dual Luciferase Assay Kit (Promega) according to the manufacturer’s instructions. The plasmid pGL3/RL or pCMV-RL was used as an internal control to calculate relative luciferase activity. Anti-HIF-1α Ab (Novus, Cat# 100–479), anti-UCHL1 Ab (Sigma-Aldrich Cat# HPA005993), anti-HA Ab (Cell Signaling Cat# 2367S), anti-Hydroxy-HIF-1α (Pro564) Ab (Cell Signaling Cat# 3434), and anti-β-actin Ab (Santa Cruz Cat# sc-69879) were used in Western blotting as primary antibodies. Anti-myc Ab (Cell Signaling Technology Cat# 2276S) was used for the immunoprecipitation of ubiquitinated HIF-1α. Anti-mouse and anti-rabbit IgG horseradish peroxidase-linked whole antibodies (GE Healthcare) were used as secondary antibodies. Amersham ECL Prime Western Blotting Detection Reagent (GE Healthcare) was used to detect chemiluminescent signals according to the manufacturer’s instructions.

### Immunoprecipitation assay

Twenty-four hours after cells (2.1 × 10^6^ HEK293T cells per 100-mm dish) were transfected with the indicated plasmids, they were harvested in 250 μL Cell Lytic Buffer (Sigma-Aldrich). The HIF-1α-myc protein was immunoprecipitated using the Immunoprecipitation Kit Dynabeads Protein G (Life Technologies) with anti-myc antibody according to the manufacturer’s instructions. Western blotting was performed using anti-HA antibody.

### Quantifications of metabolites and reduced glutathione

LC/MS-based metabolome analysis to quantify the levels of ^13^C_6_-labeled lactate, citrate, and isocitrate was performed as described previously^40^. Intracellular NADPH levels were quantified using the NADP/NADPH Quantification Colorimetric Kit (BioVision Inc.) and NADP/NADPH-Glo Assay Kit (Promega) according to the manufacturers’ instructions. Intracellular GSH levels were quantified using the Glutathione Colorimetric Assay Kit (BioVision Inc.) and GSH/GSSG-Glo Assay Kit (Promega) according to the manufacturers’ instructions.

### Clonogenic survival assay

The indicated cells (100 and 1,000 cells/60-mm dish for 0 and 2/4/8 Gy, respectively) were precultured for 24 hours with or without the indicated concentrations of NAC (4c and 4d) or 6AN (4e), treated with the indicated dose of X-radiation (Acrobio Co., Tokyo, Japan), and cultured for 2 additional weeks. NAC and 6AN were removed 24 and 1 hour after the radiation, respectively. Surviving colonies were fixed with 70% ethanol and stained with Giemsa solution. Colonies consisting of more than 50 cells were counted as surviving colonies. The plating efficiency and surviving fraction were calculated as described previously^47^.

### Statistical analyses

The significance of differences was determined using Student’s t-test. A *P*-value < 0.05 was considered to be significant.

## Electronic supplementary material

Supplementary figure S1, S2
