# Supplementary material for: UCHL1-HIF-1 axis-mediated antioxidant property of cancer cells as a therapeutic target for radiosensitization
Source: Sci Rep. 2017 Jul 31;7:6879. doi: 10.1038/s41598-017-06605-1 (PMC5537219; doi:10.1038/s41598-017-06605-1)
Supplement: Supplementary file 1 — Supplementary figure S1, S2 [file 41598_2017_6605_MOESM1_ESM.docx]

**UCHL1-HIF-1 axis-mediated antioxidant property of cancer cells as a therapeutic target for radiosensitization**

Ryota NAKASHIMA^1,2^, Yoko GOTO*^1^, Sho KOYASU^2^, Minoru KOBAYASHI^2^, Akiyo MORINIBU^2^, Michio YOSHIMURA^1^, Masahiro HIRAOKA^1^, Ester M. HAMMOND^3^, and Hiroshi HARADA^1,2,4^

^1^Department of Radiation Oncology and Image-applied Therapy, Kyoto University Graduate School of Medicine, 54 Shogoin Kawahara-cho, Sakyo-ku, Kyoto 606-8507, Japan.

^2^Laboratory of Cancer Cell Biology, Department of Genome Dynamics, Radiation Biology Center, Kyoto University, Yoshida Konoe-cho, Sakyo-ku, Kyoto 606-8501, Japan.

^3^CRUK/MRC Oxford Institute for Radiation Oncology, Department of Oncology, University of Oxford, Oxford OX3 7DQ, United Kingdom.

^4^Precursory Research for Embryonic Science and Technology (PRESTO), Japan Science and Technology (JST), 4-1-8 Honcho, Kawaguchi, Saitama 332-0012, Japan.

*Address correspondence to:

Yoko GOTO, M.D., Ph.D. Department of Radiation Oncology and Image-applied Therapy, Kyoto University Graduate School of Medicine, 54 Shogoin Kawahara-cho, Sakyo-ku, Kyoto 606-8507, Japan.

E-mail: ygoto@kuhp.kyoto-u.ac.jp


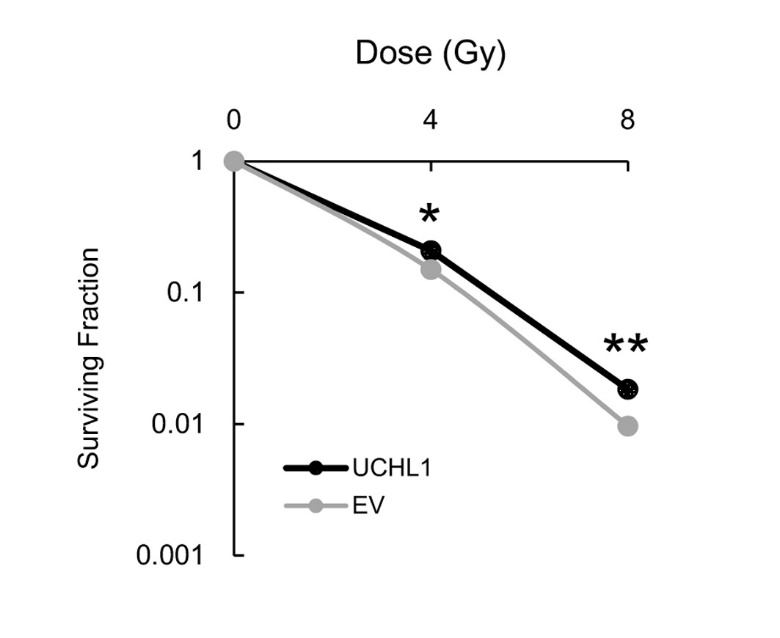


Supplementary Figure 1. The clonogenic survival assay using HeLa cells expressing UCHL1. A DNA fragment encoding UCHL1 was inserted into the BamHI-Not I site of pCDH-EF1-MCS-IRES-Puro (System Biosciences, #CD532A-2). The resultant plasmid, pCDH-EF1-UCHL1-IRES-Puro, was subjected to the lentivirus production using the pPACKH1 Lentivector Packaging Kit (System Biosciences, #LV500A-1), according to the manufacturer’s instructions. HeLa cells were infected with the lentivirus and cultured in the puromycin-containing culture medium for 8 days and treated with the indicated doses of X-radiation for the clonogenic survival assay. Means ± *s.d*. n = 3. **P*< 0.05, ***P* < 0.01 (Student’s t-test).


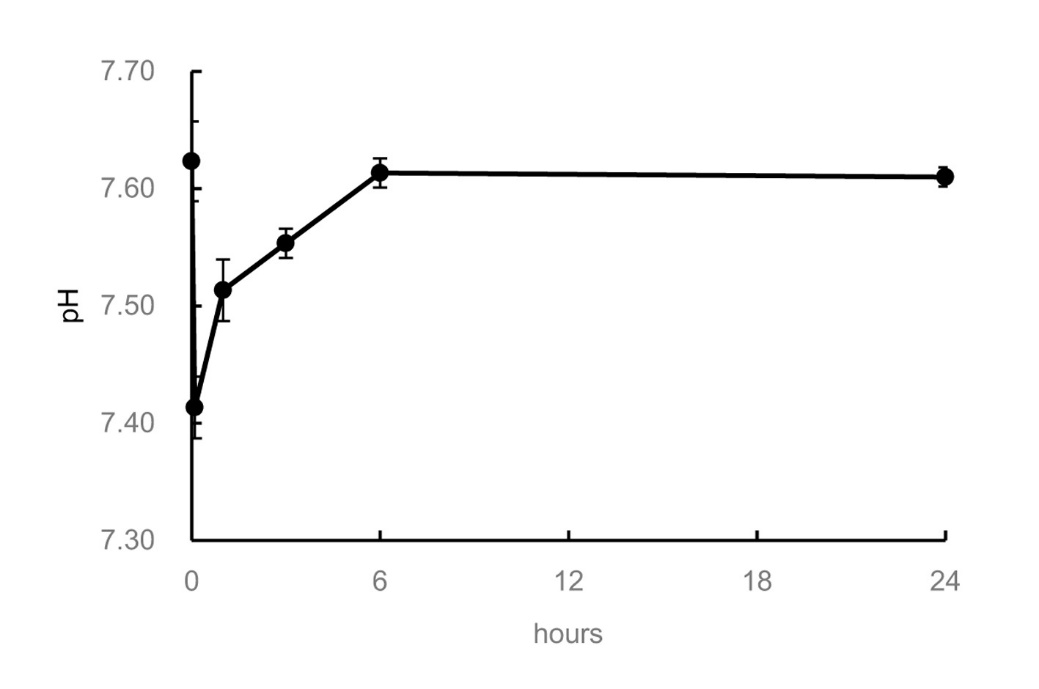


Supplementary Figure 2. Changes in pH of culture medium after adding NAC (5 mM).
